# Viscosity-Projection Method for a Family of General Equilibrium Problems and Asymptotically Strict Pseudocontractions in the Intermediate Sense

**DOI:** 10.1155/2013/608241

**Published:** 2013-10-28

**Authors:** Dao-Jun Wen

**Affiliations:** College of Mathematics and Statistics, Chongqing Technology and Business University, Chongqing 400067, China

## Abstract

In this paper, a Meir-Keeler contraction is introduced to propose a viscosity-projection approximation method for finding a common element of the set of solutions of a family of general equilibrium problems and the set of fixed points of asymptotically strict pseudocontractions in the intermediate sense. Strong convergence of the viscosity iterative sequences is obtained under some suitable conditions. Results presented in this paper extend and unify the previously known results announced by many other authors.

## 1. Introduction

Let *H* be a real Hilbert space with inner product 〈·, ·〉 and norm ||·||, respectively. Let *C* be a nonempty closed convex subset of *H*. Let *A* : *C* → *H* be a nonlinear mapping and *F* : *C* × *C* → ℝ be a bifunction, where ℝ denotes the set of real numbers. We consider the following generalized equilibrium problem: Find *x* ∈ *C* such that

(1) F(x,y)+〈Ax,y−x〉≥0, ∀y∈C.

We use *EP*⁡(*F*, *A*) to denote the set of solution of problem (1). If *A* ≡ 0, the zero mapping, then the problem (1) reduces to the normal equilibrium problem: Find *x* ∈ *C* such that

(2) F(x,y)≥0, ∀y∈C.

We use *EP*⁡(*F*) to denote the set of solution of problem (2). If *F* ≡ 0, then the problem (1) reduces to the classical variational inequality problem: Find *x* ∈ *C* such that

(3) 〈Ax,y−x〉≥0, ∀y∈C.

We use *VI*⁡(*C*, *A*) to denote the set of solution of problem (3). The generalized equilibrium problem (1) is very general in the sense that it includes, as special cases, saddle point problems, variational inequalities, optimization problems, mini-max problems, the Nash equilibrium problem in noncooperative games, and others (see, e.g., [1–4]).

Recall that a nonlinear mapping *T* : *C* → *C* is said to be nonexpansive if

(4) ||Tx−Ty||≤||x−y||, ∀x,y∈C.

*T* is said to be uniformly *L*-Lipschitz continuous if there exists a constant *L* > 0 such that

(5) ||Tnx−Tny||≤L||x−y||, n≥1,  ∀x,y∈C.

*T* is said to be asymptotically nonexpansive if there exists a sequence *k*_*n*_ ∈ [1, *∞*) with *k*_*n*_ → 1 as *n* → *∞* such that

(6) ||Tnx−Tny||≤kn||x−y||, n≥1,  ∀x,y∈C.

*T* is said to be asymptotically nonexpansive in the intermediate sense [5] if it is continuous and the following inequality holds:

(7) limsup⁡n→∞sup⁡x,y∈C(||Tnx−Tny||−||x−y||)≤0.

Putting *ξ*_*n*_ = max⁡{0, sup⁡_*x*,*y*∈*C*_(||*T* ^*n*^ *x* − *T* ^*n*^ *y*|| − ||*x* − *y*||)}, we see that *ξ*_*n*_ → 0 as *n* → *∞*. Then scheme (7) is reduced to the following:

(8) ||Tnx−Tny||≤||x−y||+ξn, ∀x,y∈C.

The class of asymptotically nonexpansive mappings in the intermediate sense was introduced by Kirk [5] as a generalization of the class of asymptotically nonexpansive mappings. It is known that, if *C* is a nonempty bounded closed convex subset of a real Hilbert space *H*, then every asymptotically nonexpansive self-mapping in the intermediate sense has a fixed point (see, e.g., [6]).

Recall also that *T* is said to be a *λ*-strict pseudocontraction [7, 8] if there exists a coefficient *λ* ∈ [0,1) such that

(9) ||Tx−Ty||2≤||x−y||2+λ||(I−T)x−(I−T)y||2,                    ∀x,y∈C.

*T* is said to be an asymptotically *λ*-strict pseudocontraction [9, 10] if there exists a sequence *k*_*n*_ ∈ [1, *∞*) with *k*_*n*_ → 1 as *n* → *∞* and a constant *λ* ∈ [0,1) such that

(10) ||Tnx−Tny||2≤kn||x−y||2+λ||(x−Tnx)−(y−Tny)  ||2,                   n≥1,  ∀x,y∈C.

*T* is said to be an asymptotically *λ*-strict pseudocontraction in the intermediate sense [11, 12] if there exists a sequence *k*_*n*_ ∈ [1, *∞*) with *k*_*n*_ → 1 as *n* → *∞* and a constant *λ* ∈ [0,1) such that

(11) limsup⁡n→∞sup⁡x,y∈C(||Tnx−Tny||2−kn||x−y||2     −λ||(x−Tnx)−(y−Tny)||2)≤0,                 ∀x,y∈C.

Putting *e*_*n*_ = max⁡{0, sup⁡_*x*,*y*∈*C*_(||*T* ^*n*^ *x*−*T* ^*n*^ *y*||^2^ − *k*_*n*_||*x*−*y*||^2^ − *λ*||(*x*−*T* ^*n*^ *x*)−(*y*−*T* ^*n*^ *y*)||^2^)}, we see that *e*_*n*_ → 0 as *n* → *∞*. Then scheme (11) is reduced to the following:

(12) ||Tnx−Tny||2  ≤kn||x−y||2+λ||(x−Tnx)−(y−Tny)||2+en,                n≥1,  ∀x,y∈C.

We use Fix⁡(*T*) to denote the set of fixed point of *T*, that is, Fix⁡(*T*) = {*x* ∈ *C* : *Tx* = *x*}. The class of asymptotically strict pseudocontractions in the intermediate sense was introduced as a generalization of the asymptotically strict pseudocontractions and asymptotically nonexpansive in the intermediate sense. Clearly, a nonexpansive mapping is a 0-strict pseudocontraction, and an asymptotically nonexpansive mapping is an asymptotically 0-strict pseudocontraction. (see, e.g., [7–12]).

Fixed point technique represent an important tool for finding the approximate solution of equilibrium problem and its variant forms, which have been studied extensively in recent years due to their applications in physics, economics, optimization, and pure and applied sciences. Some numerical methods have been proposed for finding a common element of the set of fixed point of various types of nonexpansive mappings and the set of solution of equilibrium problems with bifunctions satisfying certain conditions; see [8–20] and references therein.

In 2009, Qin et al. [10] introduced the following explicit iterative algorithm for finding a common fixed point of a finite family of asymptotically *λ*_*i*_-strictly pseudocontractions *T*_*i*_ for each *i* = 1,2,…, *N*

(13) xn=αn−1xn−1+(1−αn−1)Ti(n)h(n)xn−1, n≥1,

where *x*_0_ ∈ *C*, {*α*_*n*_}_*n*=0_^*∞*^ is a sequence in (0,1) and *n* = [*h*(*n*) − 1]*N* + *i*(*n*), *i* = *i*(*n*) = 1,2,…, *N*. They also obtain weak and strong convergence theorems based on the cyclic scheme above.

Recently, Sahu et al. [11] considered a new iterative scheme for asymptotically strictly pseudocontractive mappings in the intermediate sense. To be more precise, they proved the following theorem.

**Theorem 1 SXY**

Let *C* be a nonempty closed and convex subset of a real Hilbert space *H* and *T* : *C* → *C* be a uniformly continuous asymptotically *λ*-strictly pseudocontractive mapping in the intermediate sense with a sequence {*k*_*n*_} such that Fix⁡(*T*) is nonempty and bounded. Let {*α*_*n*_} be a sequence in [0,1] such that 0 < *δ* ≤ *α*_*n*_ ≤ 1 − *λ* for all *n* ∈ *ℕ*. Let {*x*_*n*_} ⊂ *C* be a sequence generated by the following (CQ) algorithm:

(14) u=x1∈C,yn=(1−αn)un+αnTnxn,Cn={w∈C:||yn−w||2≤||xn−w||2+θn},Qn={w∈C:〈xn−w,u−xn〉≥0},xn+1=PCn∩Qn(u),

where *θ*_*n*_ = (*k*_*n*_ − 1)*ρ*_*n*_^2^ + *e*_*n*_ and *ρ*_*n*_ = sup⁡{||*x*_*n*_ − *p*||:*p* ∈ Fix⁡(*T*)} < *∞*. Then, {*x*_*n*_} converges strongly to *P*_Fix⁡(*T*)_(*u*), where *P*_Fix⁡(*T*)_ is metric projection from *H* onto Fix⁡(*T*).

In 2011, Hu and Cai [12] modified schemes (13) and (14) to the case of asymptotically strictly pseudocontractive mappings in the intermediate sense concerning the equilibrium problem and proposed the following modified hybrid method:

(15) x0∈C, u0∈C,F(un,y)+〈Axn,y−un〉   +1rn〈y−un,un−xn〉≥0, ∀y∈C,zn=(1−βn)un+βnTi(n)h(n)un,yn=(1−αn)un+αnzn,Cn={w∈C:||yn−w||2≤||xn−w||2+θn},Qn={w∈C:〈xn−w,x0−xn〉≥0},xn+1=PCn∩Qn(x0),

where *θ*_*n*_ = (*k*_*h*(*n*)_ − 1)*ρ*_*n*_^2^ + *e*_*h*(*n*)_ → 0 as *n* → *∞* and *ρ*_*n*_ = sup⁡{||*x*_*n*_ − *p*||:*p* ∈ *Ω*} < *∞*. Moreover, they obtained convergence theorems under some suitable conditions.

On the other hand, Moudafi [13] introduced the following viscosity approximation method for fixed point problem of nonexpansive mapping

(16) xn+1=αnf(xn)+(1−αn)Txn,

where *f* is a contractive mapping. He proved that the viscosity iterative sequence {*x*_*n*_} convergence strongly to a fixed point of *T*, which is the unique solution of the variational inequality:

(17) 〈(I−f)x,z−x〉≥0, ∀z∈Fix⁡(T).

Furthermore, S. Takahashi and W. Takahashi [14] and Inchan [15] modified the viscosity approximation methods for finding a common element of the set of fixed point problems and equilibrium problems.

In 2012, Kimura and Nakajo [16] introduced a Meir-Keeler contraction and proposed a modified viscosity approximations by the shrinking projection method in Hilbert spaces, the so-called viscosity-projection method. To be more precise, they proved the following theorem.

**Theorem 1 KN**

Let *C* be a nonempty closed convex subset of *H*, and let {*T*_*n*_} be a sequence of mappings of *C* into itself with *Ω* = ⋂_*n*=1_^*∞*^Fix⁡(*T*_*n*_) ≠ *Ø* which satisfies the following condition: there exists {*a*_*n*_} ⊂ ℝ with liminf⁡_*n*→*∞*_ *a*_*n*_ > −1 such that ||*T*_*n*_ *x* − *z*||^2^ ≤ ||*x* − *z*||^2^ − *a*_*n*_||*x* − *T*_*n*_ *x*||^2^ for every *n* ∈ *ℕ*, *x* ∈ *C*, and *z* ∈ *Ω*. Let *f* be a Meir-Keeler contraction of *C* into itself, and let {*x*_*n*_} be a sequence generated by

(18) x1=x∈C,    C1=C,yn=Tnxn,Cn+1={w∈Cn:||yn−w||2≤||xn−w||2−an||xn−yn||2},xn+1=PCn+1f(xn),

for each *n* ∈ *ℕ*. For every sequence {*z*_*n*_} ⊂ *C* and *z*_*n*_ → *z* ∈ *C* and *T*_*n*_ *z*_*n*_ → *z* imply that *z* ∈ *Ω*. Then, {*x*_*n*_} converges strongly to *q* ∈ *Ω*, which satisfies *q* = *P*_*Ω*_ *f*(*q*).

In this paper, inspired and motivated by research going on in this area, we introduce a new viscosity-projection method for a family of general equilibrium problems and asymptotically strict pseudocontractions in the intermediate sense, which is defined in the following way:

(19) x1∈C, C1=C,un=FrM,nAMFrM−1,nAM−1⋯Fr2,nA2Fr1,nA1xn,zn=(1−βn)un+βnTi(n)h(n)un,yn=(1−αn)un+αnzn,Cn+1={w∈Cn:||yn−w||2≤||xn−w||2+θn},xn+1=PCn+1f(xn),

where *θ*_*n*_ = (*k*_*h*(*n*)_ − 1)*ρ*_*n*_^2^ + *e*_*h*(*n*)_ → 0 as *n* → *∞* and *ρ*_*n*_ = sup⁡{||*x*_*n*_ − *p*||:*p* ∈ *Ω*} < *∞*.

Our purpose is not only to extend the viscosity-projection method with a Meir-Keeler contraction to the case of a family of general equilibrium problems and asymptotically strict pseudocontractions in the intermediate sense, but also to obtain a strong convergence theorem by using the proposed schemes under some appropriate conditions. Results presented in this paper extend and unify the corresponding ones of [10–13, 16].

## 2. Preliminaries

Let *C* be a nonempty closed convex subset of a real Hilbert space *H* with inner product 〈·, ·〉 and norm ||·||, respectively. We use notation ⇀ for weak convergence and → for strong convergence of a sequence. For every point *x* ∈ *H*, there exists a unique nearest point in *C*, denoted by *P*_*C*_ *x*, such that

(20) ||x−PCx||≤||x−y||, ∀y∈C.

*P*_*C*_ is called the metric projection of *H* onto *C* defined by *P*_*C*_(*x*) = argmin⁡_*y*∈*C*_||*x* − *y*||. It is well known that *P*_*C*_ is nonexpansive mapping, and *u* = *P*_*C*_ *x* is equivalent to (see, e.g., [21]) the following:

(21) 〈x−u,u−y〉≥0, ∀y∈C.

Recall that a mapping *A* : *C* → *H* is said to be monotone if

(22) 〈Ax−Ay,x−y〉≥0, ∀x,y∈C.

*A* is said to be *r*-strongly monotone if there exists a constant *r* > 0 such that

(23) 〈Ax−Ay,x−y〉≥r||x−y||2, ∀x,y∈C.

*A* is said to be *α*-inverse strongly monotone if there exists a constant *α* > 0 such that

(24) 〈Ax−Ay,x−y〉≥α||Ax−Ay||2, ∀x,y∈C.

It is easy to see that if *A* is an *α*-inverse strongly monotone mapping from *C* into *H*, then *A* is 1/*α*-Lipschitz continuous.

To study the generalized equilibrium problem (1), we may assume that the bifunction *F* : *C* × *C* → ℝ satisfies the following conditions:

 (A1) *F*(*x*, *x*) = 0  for all *x* ∈ *C*;
 (A2) *F* is monotone, that is, *F*(*x*, *y*) + *F*(*y*, *x*) ≤ 0  for all *x*, *y* ∈ *C*;
 (A3) for each *x*, *y*, *z* ∈ *C*, lim⁡_*t*→0_ *F*(*tz* + (1 − *t*)*x*, *y*) ≤ *F*(*x*, *y*);
 (A4) for each *x* ∈ *C*, *y* ↦ *F*(*x*, *y*) is convex and lower semi-continuous.

**Lemma (see [1, 3])**

Let *F* : *C* × *C* → ℝ be a bifunction satisfying (A1)–(A4). Then, for any *r* > 0 and *x* ∈ *H*, there exists *z* ∈ *C* such that

(25) F(z,y)+1r〈y−z,z−x〉≥0, ∀y∈C.

Further, if *F*_*r*_ *x* = {*z* ∈ *C* : *F*(*z*, *y*) + (1/*r*)〈*y* − *z*, *z* − *x*〉≥0, ∀*y* ∈ *C*}, then the following hold:

1. *F*_*r*_ is single-valued;
2. *F*_*r*_ is firmly nonexpansive, that is, ||*F*_*r*_ *x*−*F*_*r*_ *y*||^2^ ≤ 〈*F*_*r*_ *x* − *F*_*r*_ *y*, *x* − *y*〉 for all *x*, *y* ∈ *H*;
3. Fix⁡(*F*_*r*_) = *EP*(*F*);
4. *EP*(*F*) is closed and convex.

**Lemma (see [8])**

In a Hilbert space *H*, there hold the following identities:

- ||*x*+*y*||^2^ ≤ ||*x*||^2^ + 2〈*y*, (*x* + *y*)〉, for all *x*, *y* ∈ *H*;
- ||*tx*+(1−*t*)*y*||^2^ = *t*||*x*||^2^ + (1 − *t*)||*y*||^2^ − *t*(1 − *t*)||*x*−*y*||^2^, for all *t* ∈ [0,1], for all *x*, *y* ∈ *H*.

**Lemma (see [8])**

Let *C* be a nonempty closed convex subset of a real Hilbert space *H*. For any *x*, *y*, *z* ∈ *H* and given also a real number *a* ∈ ℝ, the set

(26) {v∈C:||y−v||2≤||x−v||2+〈z,v〉+a}

is closed and convex.

**Lemma (see [11])**

Let *C* be a nonempty closed convex subset of a real Hilbert space *H* and *T* : *C* → *C* be a uniformly *L*-Lipschitz continuous and asymptotically *λ*-strict pseudocontraction in the intermediate sense. Then Fix⁡(*T*) is closed and convex.

**Lemma (see [11])**

Let *C* be a nonempty closed convex subset of a real Hilbert space *H* and *T* : *C* → *C* be a uniformly *L*-Lipschitz continuous and asymptotically *λ*-strict pseudocontraction in the intermediate sense. Then *I* − *T* is demiclosed at zero, that is, if the sequence {*x*_*n*_} ⊂ *C* such that *x*_*n*_⇀*x* and *x*_*n*_ − *Tx*_*n*_ → 0 as *n* → *∞*, then *x* ∈ Fix⁡(*T*).

**Lemma (see [11])**

Let *C* be a nonempty closed convex subset of a real Hilbert space *H* and *T* : *C* → *C* be an asymptotically *λ*-strict pseudocontraction in the intermediate sense with *γ*_*n*_ = *k*_*n*_ − 1. Then

(27) ||Tnx−Tny|| ≤11−λ{λ||x−y||      +[1+(1−λ)γn]||x−y||2+(1−λ)en},                  n≥1,  ∀x,y∈C.

Recall also that a mapping *f* of a complete metric space (*X*, *d*) into itself is called a contraction with coefficient *r* ∈ (0,1) if ||*f*(*x*) − *f*(*y*)||≤*r*||*x* − *y*||, for all *x*, *y* ∈ *X*. It is known that *f* has a unique fixed point (see, e.g., [22]). On the other hand, Meir and Keeler [23] defined the following mapping, called the Meir-Keeler contraction. A mapping *f* : *X* → *X* is called a Meir-Keeler contraction if, for every *ϵ* > 0, there exists *δ* > 0 such that *d*(*x*, *y*) < *ϵ* + *δ* implies that

(28) d(f(x),f(y))<ϵ, ∀x,y∈X.

We know that Meir-Keeler contraction is a generalization of contraction, and the following result, which extends the Banach contraction principle, is proved in [23].

**Lemma (see [23])**

A Meir-Keeler contraction defined on a complete metric space has a unique fixed point.

**Lemma (see [24])**

Let *f* be a Meir-Keeler contraction on a convex subset *C* of a Banach space *E*. Then, for every *ϵ* > 0, there exists *r* ∈ (0,1) such that ||*x* − *y*||≥*ϵ* implies that

(29) ||f(x)−f(y)||≤r||x−y||, ∀x,y∈C.

Let {*C*_*n*_} be a sequence of nonempty closed convex subsets of *H*. We define a subset *s*-*Li*_*n*_ *C*_*n*_ of *H* as follows: *x* ∈ *s*-*Li*_*n*_ *C*_*n*_ if and only if there exists {*x*_*n*_} ⊂ *H* such that *x*_*n*_ → *x* and *x*_*n*_ ∈ *C*_*n*_ for all *n* ∈ *ℕ*. Similarly, a subset *w*-*Ls*_*n*_ *C*_*n*_ of *H* is defined by *y* ∈ *w*-*Ls*_*n*_ *C*_*n*_ if and only if there exists a subsequence {*C*_*n*_*i*__} of {*C*_*n*_} and a sequence {*y*_*i*_} ⊂ *H* such that *y*_*i*_⇀*y* and *y*_*i*_ ∈ *C*_*n*_*i*__ for all *i* ∈ *ℕ*. If *C*_0_ ⊂ *H* satisfies

(30) C0=s-LinCn=w-LsnCn,

it is said that {*C*_*n*_} converges to *C*_0_ in the sense of Mosco [25], and we write *C*_0_ = *M*-lim⁡_*n*_ *C*_*n*_. One of the simplest examples of Mosco convergence is a decreasing sequence {*C*_*n*_} with respect to inclusion. The Mosco limit of such a sequence is ⋂_*n*=1_^*∞*^ *C*_*n*_. For more details, see [26].

**Lemma (see [27])**

Let {*C*_*n*_} be a sequence of nonempty closed convex subsets of *H*. If *C*_0_ = *M*-lim⁡_*n*_ *C*_*n*_ exists and is nonempty, then, for each *x* ∈ *H*, {*P*_*C*_*n*__ *x*} converges strongly to *P*_*C*_0__ *x*.

For the rest of this paper, let *F*_*m*_ : *C* × *C* → ℝ be a bifunction satisfying (A1)–(A4) and *A*_*m*_ : *C* → *H* be an *α*_*m*_-inverse strongly monotone mapping, for some *m* = 1,2,…, *M*. For each *r*_*m*_ > 0 and *x* ∈ *H*, define a mapping *F*_*r*_*m*__^*A*_*m*_^ : *H* → *C* as follows:

(31) FrmAm(x)={z∈C:Fm(z,y)+〈Amx,y−z〉  +1rm〈y−z,z−x〉≥0,  ∀y∈C}.

It follows from Lemma 1 that *F*_*r*_*m*__^*A*_*m*_^ = *F*_*r*_*m*__(*I* − *r*_*m*_ *A*_*m*_) for each *m* = 1,2,…, *M*.

Let *T*_*i*_ : *C* → *C* be a uniformly *L*_*i*_-Lipschitz continuous and asymptotically *λ*_*i*_-strict pseudocontractive mapping in the intermediate sense with the sequences {*k*_*n*,*i*_}⊂[1, *∞*) such that lim⁡_*n*→*∞*_ *k*_*n*,*i*_ = 1 and {*e*_*n*,*i*_}⊂[0, *∞*) such that lim⁡_*n*→*∞*_ *e*_*n*,*i*_ = 0, for some *i* = 1,2,…, *N*, that is,

(32) ||Tinx−Tiny||2≤kn,i||x−y||2+λi||(x−Tinx)−(y−Tiny)||2+en,i,∀x,y∈C.

Remark that *L* = max⁡{*L*_*i*_ : 1 ≤ *i* ≤ *N*}, *λ* = max⁡{*λ*_*i*_ : 1 ≤ *i* ≤ *N*}, *k*_*n*_ = max⁡{*k*_*n*,*i*_ : 1 ≤ *i* ≤ *N*, *n* ∈ *ℕ*} and *e*_*n*_ = max⁡{*e*_*n*,*i*_ : 1 ≤ *i* ≤ *N*, *n* ∈ *ℕ*}.

## 3. Main Results

**Theorem 10**

Let *C* be a nonempty closed convex subset of Hilbert space *H*. Let *F*_*m*_ : *C* × *C* → ℝ be a bifunction satisfying (A1)–(A4), and let *A*_*m*_ : *C* → *H* be an *α*_*m*_-inverse strongly monotone mapping, for each *m* = 1,2,…, *M*. Let *T*_*i*_ : *C* → *C* be a uniformly *L*_*i*_-Lipschitz continuous and asymptotically *λ*_*i*_-strict pseudocontractive mapping in the intermediate sense with the sequences {*k*_*n*,*i*_} and {*e*_*n*,*i*_} for each *i* = 1,2,…, *N*. If *f* is a Meir-Keeler contraction of *C* into itself and *Ω* = (⋂_*i*=1_^*N*^Fix⁡(*T*_*i*_))∩(⋂_*m*=1_^*M*^ *EP*(*F*_*m*_, *A*_*m*_)) is nonempty and bounded. Assume that {*α*_*n*_}, {*β*_*n*_} are sequences in [0,1] such that 0 < *a* ≤ *α*_*n*_ ≤ 1, 0 < *b* ≤ *β*_*n*_ ≤ 1 − *λ* and {*r*_*m*,*n*_}⊂(0, *∞*) such that *r*_*m*,*n*_ ∈ [*c*, *d*]⊂(0,2*α*_*m*_), for each *m* = 1,2,…, *M* and *n* ∈ *ℕ*. Then the sequence {*x*_*n*_} generated by (19) converges strongly to *q* = *P*_*Ω*_ *f*(*q*).

**Proof**

We split the proof into six steps.

*Step  1.* We prove that *P*_*Ω*_ *f* exists a unique fixed point. To do this, we first show that (*I* − *r*_*m*,*n*_ *A*_*m*_) is nonexpansive for each *m* = 1,2,…, *M*. Indeed,

(33) ||(I−rm,nAm)x−(I−rm,nAm)y||2  =||(x−y)−rm,n(Amx−Amy)||2  =||x−y||2−2rm,n〈Amx−Amy,x−y〉   +rm,n2||Amx−Amy||2  ≤||x−y||2−rm,n(2αm−rm,n)||Amx−Amy||2  ≤||x−y||2.

It follows that (*I* − *r*_*m*,*n*_ *A*_*m*_) is nonexpansive. By Lemma 1, we know that ⋂_*m*=1_^*M*^ *EP*⁡(*F*_*m*_, *A*_*m*_) is closed and convex. We also know from Lemma 4 that ⋂_*i*=1_^*N*^Fix⁡(*T*_*i*_) is closed, and convex. Hence, *Ω* = (⋂_*i*=1_^*N*^Fix⁡(*T*_*i*_))∩(⋂_*m*=1_^*M*^ *EP*⁡(*F*_*m*_, *A*_*m*_)) is a nonempty, closed and convex subset of *C*. Consequently, *P*_*Ω*_ is well-defined. Since *P*_*Ω*_ is nonexpansive, the composed mapping *P*_*Ω*_ *f* of *C* into itself is a Meir-Keeler contraction on *C*; see [24, Proposition 3]. By Lemma 7, there exists a unique fixed point *z* ∈ *Ω* of *P*_*Ω*_ *f*.

*Step  2*. We show that *C*_*n*_ is closed convex subset of *C* for each *n* ≥ 1. By the assumption of *C*_*n*+1_, it is easy to see that *C*_*n*_ is closed for each *n* ≥ 1. We only show that *C*_*n*_ is convex for each *n* ≥ 1. It is obvious that *C*_1_ = *C* is closed and convex. Suppose that *C*_*k*_ is closed and convex for some *k* ≥ 1. For any *w* ∈ *C*_*k*_, we see that

(34) ||yk−w||2≤||xk−w||2+θk, k≥1,

is equivalent to

(35) 2〈xk−yk,w〉≤||xk||2−||yk||2+θk.

Taking *w*_1_ and *w*_2_ in *C*_*k*+1_ and putting $\bar{w}=tw_{1}+(1-t)w_{2}$, it follows that *w*_1_, *w*_2_ ∈ *C*_*k*_, and so

(36) 2〈xk−yk,w1〉≤||xk||2−||yk||2+θk,

(37) 2〈xk−yk,w2〉≤||xk||2−||yk||2+θk.

Combing (36) and (37), we obtain that

(38) 2〈xk−yk,w¯〉≤||xk||2−||yk||2+θk.

That is,

(39) ||yk−w¯||2≤||xk−w¯||2+θk.

In view of the convexity of *C*_*k*_, we see that $\bar{w}\in C_{k}$. This implies that $\bar{w}\in C_{k+1}$. Therefore, *C*_*k*+1_ is convex. Consequently, *C*_*n*_ is closed and convex for each *n* ≥ 1.

*Step  3.* We show that *Ω* ⊂ *C*_*n*_ for each *n* ≥ 1. Put Θ_*n*_^*m*^ = *F*_*r*_*m*,*n*__^*A*_*m*_^ *F*_*r*_*m*−1,*n*__^*A*_*m*−1_^ ⋯ *F*_*r*_2,*n*__^*A*_2_^ *F*_*r*_1,*n*__^*A*_1_^ for every *m* = 1,2,…, *M* and Θ_*n*_^0^ = *I* for all *n* ∈ *ℕ*. Note that *F*_*r*_*m*,*n*__^*A*_*m*_^ = *F*_*r*_*m*,*n*__(*I* − *r*_*m*,*n*_ *A*_*m*_) is nonexpansive. Therefore, *u*_*n*_ = Θ_*n*_^*M*^ *x*_*n*_. It is obvious that *Ω* ⊂ *C*_1_ = *C*. Suppose that *Ω* ⊂ *C*_*k*_ for some *k* ≥ 1. Taking *p* ∈ *Ω*, it follows from Lemma 1 that

(40) ||un−p||=||ΘnMxn−ΘnMp||=||FrM,n(I−rM,nAM)ΘnM−1xn  −FrM,n(I−rM,nAM)ΘnM−1p||≤||(I−rM,nAM)ΘnM−1xn  −(I−rM,nAM)ΘnM−1p||≤||ΘnM−1xn−ΘnM−1p||⋮≤||Θn0xn−Θn0p||=||xn−p||.

From (19), we observe that

(41) ||zn−p||2=||(1−βn)un+βnTi(n)h(n)un−p||2=(1−βn)||un−p||2+βn||Ti(n)h(n)un−p||2 −βn(1−βn)||un−Ti(n)h(n)un||2≤(1−βn)||un−p||2 +βn[kh(n)||un−p||2+λ||un−Ti(n)h(n)un||2+eh(n)] −βn(1−βn)||un−Ti(n)h(n)un||2≤kh(n)||un−p||2−βn(1−βn−λ) ×||un−Ti(n)h(n)un||2+βneh(n)≤kh(n)||un−p||2+βneh(n).

By virtue of convexity of ||·||^2^, combining (40) and (41), we have

(42) ||yn−p||2 =||(1−αn)un+αnzn−p||2≤(1−αn)||un−p||2+αn||zn−p||2≤(1−αn)||un−p||2 +αn[kh(n)||un−p||2+βneh(n)]≤||un−p||2+(kh(n)−1)||un−p||2+eh(n)=||un−p||2+θn

(43)  ≤||xn−p||2+θn,

where *θ*_*n*_ = (*k*_*h*(*n*)_ − 1)*ρ*_*n*_^2^ + *e*_*h*(*n*)_ → 0 as *n* → *∞* and *ρ*_*n*_ = sup⁡{||*x*_*n*_ − *p*||:*p* ∈ *Ω*} < *∞*. Setting *n* = *k* in (40)–(43), it follows that *p* ∈ *C*_*k*+1_. Therefore, *Ω* ∈ *C*_*n*_ for each *n* ≥ 1.

*Step  4.* Next, we prove that lim⁡_*n*→*∞*_ *x*_*n*_ = *q*, where *q* = *P*_⋂_*n*=1_^*∞*^*C*_*n*__ *f*(*q*). Note that *C*_*n*_ is a closed convex subset of *H* and *∅* ≠ *Ω* ⊂ *C*_*n*+1_ ⊂ *C*_*n*_ for all *n* ∈ *ℕ*. Thus, {*x*_*n*_} is well-defined. Since the composed mapping *P*_⋂_*n*=1_^*∞*^*C*_*n*__ *f* is a Meir-Keeler contraction on *C*, there exists a unique fixed point *q* = *P*_⋂_*n*=1_^*∞*^*C*_*n*__ *f*(*q*) ∈ ⋂_*n*=1_^*∞*^ *C*_*n*_ by Lemma 7. Let *w*_*n*_ = *P*_*C*_*n*__ *f*(*q*) for each *n* ∈ *ℕ*. We get ⋂_*n*=1_^*∞*^ *C*_*n*_ = *M*-lim⁡_*n*_ *C*_*n*_, since *Ω* ⊂ *C*_*n*+1_ ⊂ *C*_*n*_ for every *n* ∈ *ℕ*. Thus, from Lemma 9, we get

(44) wn→P⋂n=1∞Cnf(q)=q.

We prove that *x*_*n*_ → *q* in the following. If it were so, it would hold that limsup⁡_*n*→*∞*_||*x*_*n*_ − *q*||>0. Let *ϵ* > 0, such that limsup⁡_*n*→*∞*_||*x*_*n*_ − *q*||>*ϵ*. By the definition of Meir-Keeler contraction, there exists *δ* > 0 with limsup⁡_*n*→*∞*_||*x*_*n*_ − *q*||>*ϵ* + *δ* such that ||*x* − *y*||<*ϵ* + *δ* implies that

(45) ||f(x)−f(y)||<ϵ, ∀x,y∈C.

From Lemma 8, there exists *r* ∈ (0,1) such that ||*x* − *y*||≥*ϵ* + *δ* implies that

(46) ||f(x)−f(y)||<r||x−y||, ∀x,y∈C.

By (44), there exists *n*_0_ ∈ *ℕ* such that ||*w*_*n*_ − *q*||<*δ* for each *n* ≥ *n*_0_. As in the proof of [24, Theorem 8], we consider the following two cases:

- There exists *n*_1_ ≥ *n*_0_ such that ||*x*_*n*_1__ − *q*||<*ϵ* + *δ*.
- ||*x*_*n*_ − *q*||≥*ϵ* + *δ* for every *n* ≥ *n*_0_.

In case (i), it holds that

(47) $$\begin{array}{c} ||x_{n_{1}+1}-w_{n_{1}+1}||\leq ||f\left(x_{n_{1}}\right)-f\left(q\right)||<\epsilon . \end{array}$$

Thus we get

(48) $$\begin{array}{c} ||x_{n_{1}+1}-q||\leq ||x_{n_{1}+1}-w_{n_{1}+1}||+||w_{n_{1}+1}-q||<\epsilon +\delta , \end{array}$$

which means that

(49) limsup⁡n→∞||xn−q||≤ϵ+δ<limsup⁡n→∞||xn−q||.

This is a contradiction. In case (ii), we have

(50) $$\begin{array}{c} ||f\left(x_{n}\right)-f\left(q\right)||<r||x_{n}-q||,\;n\geq n_{0}. \end{array}$$

Thus we get

(51) ||xn1+1−wn1+1||≤||f(xn)−f(q)||<r||xn−q||≤r(||xn−wn||+||wn−q||),

It follows from (44) that

(52) limsup⁡n→∞||xn−wn||=limsup⁡n→∞||xn1+1−wn1+1||≤r limsup⁡n→∞||xn−wn||<limsup⁡n→∞||xn−wn||.

This is a contradiction again. Therefore, we obtain that lim⁡_*n*→*∞*_ *x*_*n*_ = *q*. Moreover, since *x*_*n*+1_ = *P*_*C*_*n*+1__ *f*(*x*_*n*_), we have 〈*f*(*x*_*n*_) − *x*_*n*+1_, *x*_*n*+1_ − *y*〉≥0 for each *y* ∈ *C*_*n*+1_. By *Ω* ⊂ *C*_*n*+1_, we have

(53) 〈f(q)−q,q−y〉≥0, ∀y∈Ω,

which is equivalent to *q* = *P*_*Ω*_ *f*(*q*) from the property of metric projection.

*Step  5.* Now, we prove that lim⁡_*n*→*∞*_||*x*_*n*_ − *u*_*n*_|| = 0. From *x*_*n*_ → *q* as *n* → *∞*, we have

(54) lim⁡n→∞||xn+1−xn||=0.

Since *x*_*n*+1_ = *P*_*C*_*n*+1__ *f*(*x*_*n*_) ∈ *C*_*n*+1_, we have

(55) ||yn−xn+1||2≤||xn−xn+1||2+θn.

It follows from (55) and Lemma 2 that

(56) ||yn−xn||2=||yn−xn+1+xn+1−xn||2=||yn−xn+1||2+2〈yn−xn+1,xn+1−xn〉 +||xn+1−xn||2≤2||xn−xn+1||2+2||yn−xn+1|| ×||xn+1−xn||+θn.

Since *θ*_*n*_ = (*k*_*h*(*n*)_ − 1)*ρ*_*n*_^2^ + *e*_*h*(*n*)_ → 0 as *n* → *∞* and (54), we obtain

(57) lim⁡n→∞||xn−yn||=0.

For each *p* ∈ *Ω*, *m* = 1,2,…, *M*, it follows from (33) and (40) that

(58) ||Θnmxn−p||2 =||Frm,n(I−rm,nAm)Θnm−1xn−Frm,n(I−rm,nAm)p||2 ≤||(I−rm,nAm)Θnm−1xn−(I−rm,nAm)p||2 ≤||Θnm−1xn−p||2−rm,n(2αm−rm,n)  ×||AmΘnm−1xn−Amp||2 ≤||xn−p||2−rm,n(2αm−rm,n)  ×||AmΘnm−1xn−Amp||2.

By (40), (42), and (58), we have

(59) ||yn−p||2≤||un−p||2+θn=||ΘnMxn−ΘnMp||+θn≤||Θnmxn−Θnmp||+θn≤||xn−p||2−rm,n(2αm−rm,n) ×||AmΘnm−1xn−Amp||2+θn,

which implies that

(60) rm,n(2αm−rm,n)||AmΘnm−1xn−Amp||2  ≤||xn−p||2−||yn−p||2+θn  ≤||xn−yn||(||xn−p||+||yn−p||)+θn.

Since *r*_*m*,*n*_ ∈ [*c*, *d*]⊂(0,2*α*_*m*_) for each *m* = 1,2,…, *M* and *θ*_*n*_ → 0 as *n* → *∞*. From (57), we have

(61) lim⁡n→∞||AmΘnm−1xn−Amp||=0, m=1,2,…,M.

On the other hand, it follows from the nonexpansive *I* − *r*_*m*,*n*_ *A*_*m*_ and Lemma 1 that

(62) ||Θnmxn−p||2 =||Frm,n(I−rm,nAm)Θnm−1xn−Frm,n(I−rm,nAm)Θnm−1p||2 ≤〈(I−rm,nAm)Θnm−1xn−(I−rm,nAm)p,Θnmxn−p〉 =12[||(I−rm,nAm)Θnm−1xn−(I−rm,nAm)p||2  +||Θnmxn−p||2  −||(I−rm,nAm)Θnm−1xn−(I−rm,nAm)p−(Θnmxn−p)||2] ≤12[||Θnm−1xn−p||2+||Θnmxn−p||2  −||Θnm−1xn−Θnmxn−rm,n(AmΘnm−1xn−Amp)||2],

which implies that

(63) ||Θnmxn−p||2  ≤||Θnm−1xn−p||2   −||Θnm−1xn−Θnmxn−rm,n(AmΘnm−1xn−Amp)||2  =||Θnm−1xn−p||2−||Θnm−1xn−Θnmxn||2   −rm,n2||AmΘnm−1xn−Amp||2   +2rm,n〈Θnm−1xn−Θnmxn,AmΘnm−1xn−Amp〉  ≤||xn−p||2−||Θnm−1xn−Θnmxn||2   +2rm,n||Θnm−1xn−Θnmxn||     ·||AmΘnm−1xn−Amp||.

From (40), (42), and (63), we obtain

(64) ||yn−p||2≤||un−p||2+θn=||ΘnMxn−p||+θn≤||Θnmxn−p||+θn≤||xn−p||2−||Θnm−1xn−Θnmxn||2 +2rm,n||Θnm−1xn−Θnmxn|| ·||AmΘnm−1xn−Amp||+θn,

which implies that

(65) ||Θnm−1xn−Θnmxn||2 ≤||xn−p||2−||yn−p||2+2rm,n||Θnm−1xn−Θnmxn||  ×||AmΘnm−1xn−Amp||+θn ≤||xn−yn||(||xn−p||+||yn−p||)  +2rm,n||Θnm−1xn−Θnmxn||||AmΘnm−1xn−Amp||+θn.

It follows from (57) and (65) that

(66) lim⁡n→∞||Θnm−1xn−Θnmxn||=0, m=1,2,…,M.

Note that

(67) ||xn−un||≤||Θn0xn−Θn1xn||+||Θn1xn−Θn2xn||+⋯+||ΘnM−1xn−ΘnMxn||.

Therefore,

(68) lim⁡n→∞||xn−un||=0.

*Step  6.* Finally, we prove that *q* ∈ (⋂_*i*=1_^*N*^Fix⁡(*T*_*i*_))∩(⋂_*m*=1_^*M*^ *EP*⁡(*F*_*m*_, *A*_*m*_)). To do this, we first show that *q* ∈ ⋂_*i*=1_^*N*^Fix⁡(*T*_*i*_). Note that

(69) ||un+1−un||≤||un+1−xn+1||+||xn+1−xn||+||xn−un||.

From (54) and (68), we get

(70) lim⁡n→∞||un+1−un||=0.

It follows that

(71) lim⁡n→∞||un+i−un||=0, i=1,2,…,N.

For any positive integer *n* ≥ *N*, note that *n* = [*h*(*n*) − 1]*N* + *i*(*n*), where *i* = *i*(*n*) = 1,2,…, *N*. By (19) and the conditions 0 < *a* ≤ *α*_*n*_ ≤ 1 and 0 < *b* ≤ *β*_*n*_ ≤ 1 − *λ*, we have

(72) ||un−Ti(n)h(n)un||=1βn||zn−un||=1αnβn||yn−un||≤1ab(||yn−xn||+||xn−un||).

From (57) and (68), we get

(73) lim⁡n→∞||un−Ti(n)h(n)un||=0.

By the fact that *h*(*n*) = *h*(*n* − *N*) + 1 and *i*(*n*) = *i*(*n* − *N*), we observe that

(74) ||un−Ti(n)h(n)−1un||≤||un−un−N|| +||un−N−Ti(n−N)h(n−N)un−N|| +||Ti(n−N)h(n−N)un−N−Ti(n)h(n)−1un||.

Applying (71), (73), and Lemma 6, we obtain

(75) lim⁡n→∞||un−Ti(n)h(n)−1un||=0.

By the uniformly *L*-Lipschitzian of *T*_*i*_, we have

(76) ||un−Tnun||≤||un−Ti(n)h(n)un||+||Ti(n)h(n)un−Ti(n)un||≤||un−Ti(n)h(n)un||+L||un−Ti(n)h(n)−1un||.

It follows from (73) and (75) that

(77) lim⁡n→∞||un−Tnun||=0.

Since

(78) ||un−Tn+iun||≤||un−un+i||+||un+i−Tn+iun+i||+||Tn+iun+i−Tn+iun||≤(1+L)||un−un+i||+||un+i−Tn+iun+i||.

Combining (71) and (77), we obtain

(79) lim⁡n→∞||un−Tiun||=0, i=1,2,…,N.

Moreover, for each *i* = 1,2,…, *N*, we have

(80) ||xn−Tixn||≤||xn−un||+||un−Tiun||+||Tiun−Tixn||.

This implies that

(81) lim⁡n→∞||xn−Tixn||=0, i=1,2,…,N.

Note that *x*_*n*_ → *q* as *n* → *∞*. It follows from (81) and Lemma 5 that *q* ∈ ⋂_*i*=1_^*N*^Fix⁡(*T*_*i*_).

Next, we show that *q* ∈ ⋂_*m*=1_^*M*^ *EP*⁡(*F*_*m*_, *A*_*m*_). From Lemma 1 and since Θ_*n*_^*m*^ *x*_*n*_ = *F*_*r*_*m*,*n*__(*I* − *r*_*m*,*n*_ *A*_*m*_)Θ_*n*_^*m*−1^ *x*_*n*_, *m* = 1,2,…, *M*, we have

(82) Fm(Θnmxn,y)+〈AmΘnm−1xn,y−Θnmxn〉   +1rm,n〈y−Θnmxn,Θnmxn−Θnm−1xn〉≥0,                    ∀y∈C.

By (A2), we have

(83) 〈AmΘnm−1xn,y−Θnmxn〉   +1rm,n〈y−Θnmxn,Θnmxn−Θnm−1xn〉  ≥Fm(y,Θnmxn), ∀y∈C.

Let *z*_*t*_ = *ty* + (1 − *t*)*q* for all *t* ∈ (0,1] and *y* ∈ *C*. This implies that *z*_*t*_ ∈ *C*. Then, we have

(84) 〈Amzt,zt−Θnmxn〉  ≥〈Amzt,zt−Θnmxn〉−〈AmΘnm−1xn,zt−Θnmxn〉   −〈zt−Θnmxn,Θnmxn−Θnm−1xnrm,n〉+Fm(zt,Θnmxn)  ≥〈Amzt−AmΘnmxn,zt−Θnmxn〉   +〈AmΘnmxn−AmΘnm−1xn,zt−Θnmxn〉   −〈zt−Θnmxn,Θnmxn−Θnm−1xnrm,n〉+Fm(zt,Θnmxn).

From (66), we have ||*A*_*m*_Θ_*n*_^*m*^ *x*_*n*_ − *A*_*m*_Θ_*n*_^*m*−1^ *x*_*n*_||→0 as *n* → *∞*. Moreover, by (A4) and the monotonicity of *A*_*m*_, we obtain

(85) 〈Amzt,zt−q〉≥Fm(zt,q), m=1,2,…,M.

Using (A1), (A4), and (85), we obtain

(86) 0=Fm(zt,zt)≤tFm(zt,y)+(1−t)Fm(zt,q)≤tFm(zt,y)+(1−t)〈Amzt,zt−q〉≤tFm(zt,y)+(1−t)t〈Amzt,y−q〉.

and hence

(87) Fm(zt,y)+(1−t)〈Amzt,y−q〉≥0.

Let *t* → 0, from (A3) and (87), we have

(88) Fm(q,y)+〈Amq,y−q〉≥0, ∀y∈C,  m=1,2,…,M.

This implies that *q* ∈ *EP*⁡(*F*_*m*_, *A*_*m*_), *m* = 1,2,…, *M*. Therefore, *q* ∈ ⋂_*m*=1_^*M*^ *EP*⁡(*F*_*m*_, *A*_*m*_). Consequently, we obtain that *q* ∈ *Ω*. This completes the proof.

We also obtain the following results by using the viscosity-hybrid projection methods, which extend and improve the hybrid method (CQ) proposed by Sahu et al. [11] and Hu and Cai [12].

**Theorem**

Let *C* be a nonempty closed convex subset of Hilbert space *H*. Let *F*_*m*_ : *C* × *C* → ℝ be a bifunction satisfying (A1)–(A4), and let *A*_*m*_ : *C* → *H* be an *α*_*m*_-inverse strongly monotone mapping, for each *m* = 1,2,…, *M*. Let *T*_*i*_ : *C* → *C* be a uniformly *L*_*i*_-Lipschitz continuous and asymptotically *λ*_*i*_-strict pseudocontractive mapping in the intermediate sense with the sequences {*k*_*n*,*i*_} and {*e*_*n*,*i*_} for each *i* = 1,2,…, *N*. If *f* is a Meir-Keeler contraction of *C* into itself and *Ω* = (⋂_*i*=1_^*N*^Fix⁡(*T*_*i*_))∩(⋂_*m*=1_^*M*^ *EP*⁡(*F*_*m*_, *A*_*m*_)) is nonempty and bounded. Let {*x*_*n*_} be a sequence defined by

(89) x1∈C, Q1=C,un=FrM,nAMFrM−1,nAM−1⋯Fr2,nA2Fr1,nA1xn,zn=(1−βn)un+βnTi(n)h(n)un,yn=(1−αn)un+αnzn,Cn={w∈C:||yn−w||2≤||xn−w||2+θn},Qn={u∈Qn−1:〈f(xn−1)−xn,xn−u〉≥0},xn+1=PCn⋂Qnf(xn),

where *θ*_*n*_ = (*k*_*h*(*n*)_ − 1)*ρ*_*n*_^2^ + *e*_*h*(*n*)_ → 0 as *n* → *∞* and *ρ*_*n*_ = sup⁡{||*x*_*n*_ − *p*||:*p* ∈ *Ω*} < *∞*. Assume that {*α*_*n*_} and {*β*_*n*_} are sequences in [0,1] such that 0 < *a* ≤ *α*_*n*_ ≤ 1, 0 < *b* ≤ *β*_*n*_ ≤ 1 − *λ* and {*r*_*m*,*n*_}⊂(0, *∞*) such that *r*_*m*,*n*_ ∈ [*c*, *d*]⊂(0,2*α*_*m*_), for each *m* = 1,2,…, *M*. Then the sequence {*x*_*n*_} generated by (89) converges strongly to *q* = *P*_*Ω*_ *f*(*q*).

**Proof**

We have that *C*_*n*_ and *Q*_*n*_ are closed convex subsets of *H* and *Ω* ⊂ *C*_*n*_ for every *n* ∈ *ℕ*. We only prove that *Ω* ⊂ *Q*_*n*_ for every *n* ∈ *ℕ* and that a sequence {*x*_*n*_} is well-defined. We have *x*_1_ ∈ *C* and *Ω* ⊂ *Q*_1_ = *C*. Assume that *x*_*k*_ ∈ *C* and *Ω* ⊂ *Q*_*k*_ for some *k* ∈ *ℕ*. Since *Ω* ⊂ *C*_*k*_∩*Q*_*k*_, there exists a unique element *x*_*k*+1_ = *P*_*C*_*k*_∩*Q*_*k*__ *f*(*x*_*k*_), and hence

(90) 〈f(xk)−xk+1,xk+1−y〉≥0, ∀y∈Ck∩Qk.

This implies that

(91) 〈f(xk)−xk+1,xk+1−y〉≥0, ∀y∈Ω.

That is, *Ω* ⊂ *Q*_*k*+1_. Therefore, we prove that *Ω* ⊂ *Q*_*n*_.

On the other hand, *P*_⋂_*n*=1_^*∞*^*Q*_*n*__ *f* is a Meir-Keeler contraction on *C*, there exists a unique element *q* = *P*_⋂_*n*=1_^*∞*^*Q*_*n*__ *f*(*q*) ∈ ⋂_*n*=1_^*∞*^ *Q*_*n*_ by Lemma 7. Let *z*_*n*_ = *P*_*Q*_*n*__ *f*(*q*) for each *n* ∈ *ℕ*. Since *Ω* ⊂ *Q*_*n*+1_ ⊂ *Q*_*n*_, it follows from Lemma 9 that *z*_*n*_ → *q* = *P*_⋂_*n*=1_^*∞*^*Q*_*n*__ *f*(*q*). We also have *x*_*n*_ = *P*_*Q*_*n*__ *f*(*x*_*n*−1_) by the definition of *Q*_*n*_. Therefore, as in the proof of Theorem 10, we get *x*_*n*_ → *q*, and the desired conclusion follows immediately from Theorem 10. This completes the proof.

If *M* = *N* = 1, we obtain the following corollary for a general equilibrium problem and asymptotically strict pseudocontraction in the intermediate sense as a special cases.

**Theorem 12**

Let *C* be a nonempty closed convex subset of Hilbert space *H*. Let *F* : *C* × *C* → ℝ be a bifunction satisfying (A1)–(A4) and *A* : *C* → *H* be an *α*-inverse strongly monotone mapping. Let *T* : *C* → *C* be a uniformly *L*-Lipschitz continuous and asymptotically *λ*-strict pseudocontractive mapping in the intermediate sense with the sequences {*k*_*n*_} and {*e*_*n*_}. If *f* is a Meir-Keeler contraction of *C* into itself and *Ω* = Fix⁡(*T*)∩*EP*⁡(*F*, *A*) is nonempty and bounded. Let {*x*_*n*_} be a sequence defined by

(92) x1∈C, C1=C,F(un,y)+〈Axn,y−un〉   +1rn〈y−un,un−xn〉≥0, ∀y∈C,zn=(1−βn)un+βnTnun,yn=(1−αn)un+αnzn,Cn+1={w∈Cn:||yn−w||2≤||xn−w||2+θn},xn+1=PCn+1f(xn),

where *θ*_*n*_ = (*k*_*n*_ − 1)*ρ*_*n*_^2^ + *e*_*n*_ → 0 as *n* → *∞* and *ρ*_*n*_ = sup⁡{||*x*_*n*_ − *p*|| : *p* ∈ *Ω*} < *∞*. Assume that {*α*_*n*_} and {*β*_*n*_} are sequences in [0,1] such that 0 < *a* ≤ *α*_*n*_ ≤ 1, 0 < *b* ≤ *β*_*n*_ ≤ 1 − *λ* and {*r*_*n*_}⊂(0, *∞*) such that *r*_*n*_ ∈ [*c*, *d*]⊂(0,2*α*). Then the sequence {*x*_*n*_} generated by (92) converges strongly to *q* = *P*_*Ω*_ *f*(*q*).

If *M* = *N* = 1 and *F* = 0, the general equilibrium problem (1) reduces into the classical variational inequality problem (3), we obtain the following corollary as a special case of Theorems 10 and 12.

**Theorem 13**

Let *C* be a nonempty closed convex subset of Hilbert space *H*. Let *A* : *C* → *H* be an *α*-inverse strongly monotone mapping. Let *T* : *C* → *C* be a uniformly *L*-Lipschitz continuous and asymptotically *λ*-strict pseudocontractive mapping in the intermediate sense with the sequences {*k*_*n*_} and {*e*_*n*_}. If *f* is a Meir-Keeler contraction of *C* into itself and *Ω* = Fix⁡(*T*)∩*VI*(*C*, *A*) is nonempty and bounded. Let {*x*_*n*_} be a sequence defined by

(93) x1∈C, C1=C,yn=(1−αn)xn+αnTnPC(xn−rnAxn),Cn+1={w∈Cn:||yn−w||2≤||xn−w||2+θn},xn+1=PCn+1f(xn),

where *θ*_*n*_ = (*k*_*n*_ − 1)*ρ*_*n*_^2^ + *e*_*n*_ → 0 as *n* → *∞* and *ρ*_*n*_ = sup⁡{||*x*_*n*_ − *p*|| : *p* ∈ *Ω*} < *∞*. Assume that {*α*_*n*_} is a sequence in [0,1] such that 0 < *b* ≤ *α*_*n*_ ≤ 1 − *λ* and {*r*_*n*_}⊂(0, *∞*) such that *r*_*n*_ ∈ [*c*, *d*]⊂(0,2*α*). Then the sequence {*x*_*n*_} generated by (93) converges strongly to *q* = *P*_*Ω*_ *f*(*q*).

**Proof**

If *F* = 0, the general equilibrium problem (1) reduces into the classical variational inequality problem (3), and

(94) 〈Axn,y−un〉+1rn〈y−un,un−xn〉≥0, ∀y∈C,

which is equivalent to

(95) 〈y−un,un−(xn−rnAxn)〉≥0, ∀y∈C.

Therefore, we have *u*_*n*_ = *P*_*C*_(*x*_*n*_ − *r*_*n*_ *Ax*_*n*_). The desired conclusion follows immediately from Theorem 10 (Set *α*_*n*_ = 1, *β*_*n*_ = *α*_*n*_). This completes the proof.
